# Evaluation of bone marrow mononuclear cells as an adjunct therapy to minced muscle graft for the treatment of volumetric muscle loss injuries

**DOI:** 10.1186/s13287-017-0589-z

**Published:** 2017-06-09

**Authors:** Stephen M. Goldman, Beth E. P. Henderson, Benjamin T. Corona

**Affiliations:** United States Army Institute of Surgical Research, 3698 Chambers Pass, Bldg. 3611, Fort Sam Houston, TX 78234 USA

**Keywords:** Bone marrow mononuclear cells, Volumetric muscle loss, Skeletal muscle, Regenerative medicine, Minced muscle graft, Musculoskeletal trauma

## Abstract

**Background:**

The delivery of alternative myogenic cell sources to enhance the efficacy of minced muscle grafts (MG) for the treatment of volumetric muscle loss (VML) injuries is a promising strategy to overcome the demand on muscle-derived donor tissue that currently limits the translation of this therapy.

**Methods:**

Using a rat model of VML, bone marrow mononuclear cells (BMNCs) were evaluated for their ability to directly contribute to de novo muscle fiber regeneration by transplanting MG in a collagen carrier at a dose of 50% of the VML injury both with and without concomitant delivery of 5 million BMNCs derived via density gradient centrifugation from the bone marrow of a syngeneic green fluorescent protein (GFP)^+^ donor.

**Results:**

Histological, molecular, and functional analyses revealed that BMNCs can engraft with co-delivered MG and contribute to nascent myofiber, but do so at a low magnitude without resulting in significant changes to transcription of key myogenic genes or gains in whole muscle force generation relative to MG alone.

**Conclusion:**

As such, co-delivery of BMNCs with MG is a promising treatment paradigm to VML that will require further investigation to identify the phenotype and therapeutic dosing of the bone marrow-derived cell populations which engraft most efficiently.

## Background

Volumetric muscle loss (VML) is a condition characterized by the loss of tissue beyond the endogenous regenerative capacity of the affected musculature and the associated persistent functional deficit for which there is no current standard of care [1]. Transplantation of minced muscle grafts (MG) is a potential therapy for some VML indications that has been shown to be rich in pax7^+^ satellite cells and effective in promoting meaningful de novo fiber regeneration at doses ≥50% of the tissue lost due to injury [2–4]. Furthermore, MG falls under the US Food & Drug Administration’s definition of a minimally manipulated tissue, and thus represents a near-term solution. Clinically, the demands on muscle autograft become a limiting factor for cases presenting large or multiple injuries. While pax7^+^ satellite cells are an absolute requirement for de novo myofiber regeneration [5], alternative cell types are capable of contributing to nascent myofibers in the presence of satellite cells [6–9]. As such, delivery of nonmuscle-derived autologous stem cells shows promise as an adjunct therapy to enhance the regenerative capacity of MG, thereby diminishing sourcing limitations and raising the possibility of treating larger VML injuries.

Bone marrow is a rich source of stem cells and has a well-developed history in transplant medicine. Bone marrow-derived cells are expected to promote healing by multiple effector functions including the production of growth factors, pro-inflammatory cytokines, and anti-inflammatory cytokines which stimulate cell proliferation, inhibit apoptosis, recruit cells, reduce fibrosis, and induce angiogenesis [10, 11]. Moreover, unfractionated bone marrow cells have been shown to participate in muscle regeneration in a recoverable cardiotoxin-induced injury model [12], and to enhance minced graft-mediated myogenesis in coculture implants in the peritoneal cavity of mice, quadriceps in the rat, and monkey arm VML injuries [13–16]. While these results are promising, further fractionation of bone marrow cells to isolate the mononuclear fraction, consisting of B cells (~50%), monocytes/dendritic cells (~5%), other CD45^+^ cells (e.g., CD34^+^CD105^–^ hematopoeitic stem cells) and a variety of CD45^–^ cells (e.g., CD45^–^CD105^+^ mesenchymal stem cells), is expected to result in greater myogenic efficacy when delivered in combination with a MG therapy [17]. This expectation is based on the fact that delivery of the bone marrow mononuclear cell (BMNC) fraction enriches the therapy for the resident stem cell populations of the bone marrow and removes red blood cells and platelets which might deleteriously impact the efficacy of said stem cells [18]. Furthermore, BMNCs have also been shown to improve skeletal muscle function, as case reports have highlighted improved muscle function following BMNC transplantation in children with Duchenne muscular dystrophy [19, 20]. It is for these reasons, and the fact that separation of the bone marrow into various cell fractions is clinically mature and relatively simple to perform, that we sought to evaluate BMNCs as an adjunct therapy to MG.

**Fig. 1:**
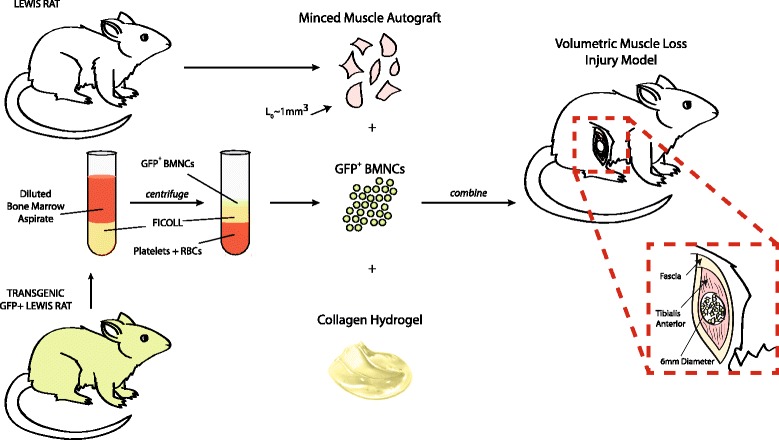
Using a rat model of VML, we tested the hypothesis that bone marrow mononuclear cells (*BMNCs*) directly contribute to de novo muscle fiber regeneration, by transplanting MG in a collagen carrier at a dose of 50% of the VML injury both with and without concomitant delivery of 5 million BMNCs derived via density gradient centrifugation from the bone marrow of a syngeneic greens fluorescent protein (*GFP*)^+^ donor. *RBC* red blood cell

In this study, we used a rat model of VML to test the hypothesis that BMNCs directly contribute to de novo muscle fiber regeneration by transplanting MG in a collagen carrier at a dose of 50% of the VML injury both with and without concomitant delivery of 5 million BMNCs derived via density gradient centrifugation from the bone marrow of a syngeneic green fluorescent protein (GFP) expressing donor (Fig. 1). Biochemical and immunofluorescence analyses were performed at 2 and 8 weeks with in vivo assessment of muscle function at 8 weeks.

## Materials and methods

### VML model

Under anesthesia (1–3% isoflurane) and in sterile conditions, a lateral incision was made through the skin lengthwise along the lateral aspect of the tibialis anterior (TA) muscle of the left hind limb of adult male Lewis rats (Harlan Laboratories, Indianapolis, IN, USA). After reflecting the skin and fascia from the anterior surface, the TA muscle and underlying extensor digitorum longus (EDL) muscle were separated by blunt dissection. A metal plate was inserted between the muscles and a 6-mm biopsy punch was used to excise a ~20% volume defect, and MG constructs were delivered acutely to the VML wound bed. The wound was closed in layers by suturing (fascia) and stapling (skin).

### BMNC isolation

Bone marrow was flushed from the intramedullary space of the long bones from transgenic GFP-expressing Lewis rats (Lew-Tg(CAG-EGFP), Rat Resource & Research Center, Columbia, MO, USA), and diluted to a total volume of 4 mL with Hank’s Balanced Salt Solution. BMNCs were isolated from the diluted bone marrow aspirate on a Ficoll (3 mL) density gradient (Ficoll-Paque™ PLUS, 1.077 g/mL; GE Healthcare, Buckinghamshire, UK) after unbraked centrifugation (400 g) at room temperature for 40 min. After washing twice, viable BMNC yield was assessed via Trypan Blue exclusion and automated cell counter (Countess™, Invitrogen, Carlsbad, CA, USA).

### Construct preparation

MG were derived from the TA muscle of a syngeneic wild-type Lewis rat. The excised muscle tissue was minced into ~1 mm^3^ pieces. After mincing, MG at a dose equal to approximately 50% of the frank loss of tissue was mixed into a collagen (400 μL, 3 mg/mL) solution with or without 5 million BMNCs (dose based on single donor BMNC yield) and cast within the wells of a 48-well cell culture plate. The collagen constructs were then allowed to crosslink at 37 °C for 30 min, after which time the constructs were transplanted to the VML defect.

### In vivo functional assessments

With the animal in a supine position, the knee of the rat was secured in place at 90° by a clamp apparatus. The foot was strapped to a boot-like foot pedal with the ankle at a 90° angle connected to a servomotor controlled force-displacement transducer (Aurora Scientific, Aurora, ON, USA). The peroneal nerve was stimulated using percutaneous needle electrodes placed around the peroneal nerve. Optimal voltage was determined using a series of twitch and tetanic contractions. Contractile function of the TA muscle was assessed by first severing the distal tendon of the synergist EDL muscle and measuring peak isometric torque as a function of stimulation frequency (400 ms train; 0.1 ms pulse width; 1–10 V; 10–200 Hz). The servomotor input and force and displacement transducer outputs are controlled and acquired, respectively, using a PC equipped with a data acquisition board (National Instruments) and custom designed Lab View (National Instruments) based software program.

### Transcriptional analysis

A portion of the defect region of the VML injury was collected at 2 and 8 weeks post-injury and immediately snap frozen in liquid nitrogen. Reverse transcription quantitative polymerase chain reaction (RT-qPCR) was used to quantify gene expression within the VML defect region. RNA was isolated from the homogenized tissue using the TRIzol method. The RNA samples were reverse transcribed into cDNA using a QuantiTech Rev Transcription kit (Qiagen, Hilden, Germany) according to the manufacturer’s protocol. Gene expression for target markers was determined using custom-designed primers for embryonic myosin heavy chain (eMHC, forward 5′-TGGAGGACCAAATATGAGACG-3′; reverse 5′-CACCATCAAGTCCTCCACCT-3′) and myogenin (forward 5′-CTACAGGCCTTGCTCAGCTC-3′; reverse 5′-GTTGGGACCAAACTCCAGTG-3′) with RT-qPCR amplification performed in the presence of SYBR Green (Bio-Rad Laboratories, Hercules, CA, USA). The raw fluorescence data were processed using LinRegPCR (v12.11; http://www.hartfaalcentrum.nl) with glyceraldehyde-3-phosphate dehydrogenase (GAPDH) serving as the endogenous control. Expression (*n* = 4–6 per group) of each target gene was calculated relative to samples from the contralateral limb.

### Immunofluorescence analysis

A portion of the neotissue from the defect region was embedded in a talcum-based gel, frozen in 2-methylbutane, and supercooled in liquid nitrogen. Cryosections (8 μm) were prepared and probed for laminin (1:100; catalog ab34360; Abcam) and GFP (1:100; catalog ab6673; Abcam) and detected with fluorescent antibodies (1:200; catalog A11055 and A21207; Invitrogen) to assess direct myogenic contribution of BMNCs. Qualitative assessments were made by observing three sections (separated by no less than 160 μm) from five muscles per group.

### Statistical analysis

Data are reported as the mean ± SEM with statistically significant differences defined as *p* < 0.05 using two-way analysis of variance (ANOVA) with Tukey’s post-hoc tests for multiple comparisons. Sample sizes for gene expression and in vivo neuromuscular strength assessments are *n* = 4–6 per group and time point.

## Results

**Fig. 2:**
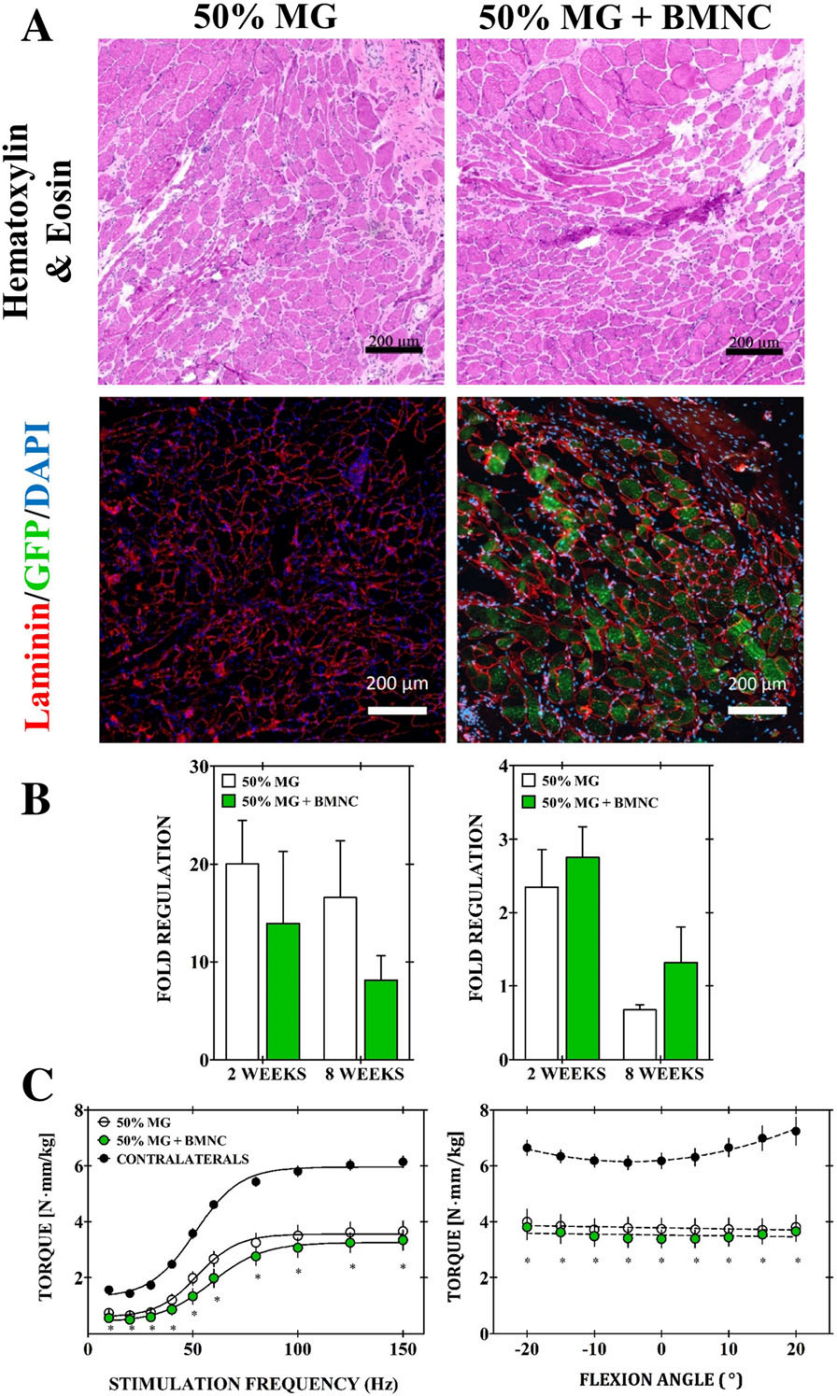
Bone marrow mononuclear cells (*BMNCs*) definitively contribute to de novo muscle fiber regeneration, but do not promote transcriptional or functional gains beyond those provided by minced muscle graft (*MG*). **a** Representative hematoxylin and eosin sections and immunofluorescence staining of green fluorescent protein (GFP)^+^ muscle fibers in the defect regions of 50% MG + BMNC (*right*) and 50% MG treated VML injuries (*left*) in wild-type hosts at 8 weeks post-injury. **b** Transcription of myogenesis markers, embryonic myosin heavy chain (*eMHC*) and *myogenin,* was not significantly different between BMNC supplemented and unsupplemented MG repair groups for either of the time points investigated (*p* > 0.05). **c** BMNC contribution to myofiber regeneration did not result in a change in in vivo isometric tetanic force generation of treated TA muscles at 8 weeks post-injury for any of the flexion angles tested. Values are shown as means ± SEM. *p < 0.05, 50% MG and 50% MG + BMNC groups versus contralateral limb

A BMNC subpopulation definitively contributed to muscle fiber regeneration as evidenced by immunofluorescence staining of GFP^+^ muscle fibers in the defect regions of VML-injured wild-type hosts (Fig. 2a). The overall direct contribution to myofiber regeneration, however, was low and localized to small regions within the broader defect area which was otherwise filled with what are assumed to be MG derived neofibers and extracellular matrix based on prior observations [3]. This observation was supported on a transcriptional level, as no significant difference was measured between the 50% MG and 50% MG with BMNC groups for the expression of the genes comprising our myogenic panel (*eMHC, myogenin*) at either of the time points investigated (Fig. 2b). Furthermore, BMNC contribution to muscle fiber regeneration does not result in an appreciable increase in the force generation of VML-injured muscle. There was no observed difference in peak isometric torque between BMNC supplemented and MG repair groups at the 8-week time point (Fig. 2c). Force-frequency curves for the two groups were statistically similar across all stimulation frequencies and joint angles with similar peak isometric torque deficits of 44.6 ± 6.1% (MG) and 45.6 ± 6.7% (MG + BMMC) relative to the unaffected contralateral control limbs.

## Discussion

While the initial goal of identifying an autologous cell source requiring minimal manipulation prior to usage as an adjunct therapy to enhance the therapeutic effect of MG on VML was not achieved, the findings of this study provide strong justification for further pursuit of a bone marrow-derived stem cell source for supplementation of MG therapy to VML. Some portion of the heterogeneous BMNC population delivered herein contributed to myofiber regeneration in agreement with previous findings [12]. What was not determined, however, was which subgroups of the greater BMNC population are responsible for the observed engraftment. Studies by Sherwood et al. [8] and Polesskaya et al. [9] independently evaluated the myogenic capacity of these bone marrow-derived cell types and produced conflicting reports with respect to capacity for myogenic differentiation of the non-hematopoietic (CD45^–^) and hematopoietic (CD45^+^) stem cell populations. Given these reports, the most prudent path forward for an autologous cellular supplement to MG would be to evaluate serial depletions of the BMNC population. Furthermore, fractionation would allow the assessment of both of these myogenic stem cell populations without the added complication of the immune cell populations which might drive a fibrotic rather than myogenic response [21, 22]. While not as clear cut as using freshly isolated BMNCs from a regulatory standpoint, the approach of using a fractionated autologous BMNC subpopulation is promising due to the maturity of immunomagentic separation as a clinical bioprocess [23–28].

A second hurdle to efficacy for this approach may reside in the delivery of a clinically impactful dose of BMNC-derived stem cells. This study focused on a single therapeutic dose of BMNCs (5 million cells per construct) freshly isolated via density gradient centrifugation, which did not result in an improvement in the functional capacity of the treated TA muscle relative to MG treatment alone. This BMNC dose and isolation methodology were chosen based on harvesting limitations of a single donor and the desire to utilize standard point of-care methodologies with a emphasis on clinical practicability. This dose, however, may vary in stem cell composition due to heterogeneity of both cell phenotype and composition. Furthermore, there is conflicting literature about the impact of density gradient centrifugation, and particularly the use of Ficoll as a medium, as an isolation methodology on the yield and potency of the resultant BMNC population [17, 29]. As such, these factors represent opportunities for optimization once depletion studies have elicited a candidate BMNC subpopulation for enrichment. It is possible that the optimal dose of BMNC-derived stem cells necessary to achieve meaningful gains in functional capacity may be superphysiological. This would necessitate an efficient isolation and ex vivo expansion methodologies to maximize yield of the effector stem cell populations or require a shift from an autologous to an allogenic approach. Both approaches face their own challenges, as ex vivo expansion raises additional manufacturing and regulatory challenges, while allogenic sourcing raises the possibility of originating graft-versus-host disease. As such, translation of either of these approaches would require further efforts to ensure their safety and feasibility. The benefit of a successful cellular supplement to MG therapy for VML would certainly justify further research into these approaches.

## Conclusions

MG represents a promising solution to VML, a clinical problem with no current standard of care. MG, however, is reliant on sufficient autologous tissue to source the grafts. If this hurdle to clinical practice is to be overcome, supplemental therapies such as the concomitant delivery of BMNCs to the affected musculature will become necessary to achieve restoration of functional muscle tissue. While the results reported herein remain suboptimal, they present a promising treatment paradigm that is ripe for investigation and further development. Success of this paradigm will likely require meticulous lineage depletion studies to identify which BMNC subpopulations to include in an adjunct therapy and at what relative magnitude with respect to a given therapeutic MG dose. If this success is achieved, it is likely to have a significant clinical impact on the treatment of traumatic orthopedic wounds.

## Abbreviations

BMNC: Bone marrow mononuclear cell
EDL: Extensor digitorum longus
GAPDH: Glyceraldehyde-3-phosphate dehydrogenase
GFP: Green fluorescent protein
MG: Minced muscle graft
RT-qPCR: Reverse transcriptase quantitative polymerase chain reaction
TA: Tibialis anterior
VML: Volumetric muscle loss
